# Intra-operative radiological diagnosis of a tip roll-over electrode array displacement using fluoroscopy, when electrophysiological testing is normal: the importance of both techniques in cochlear implant surgery^☆^

**DOI:** 10.1016/j.bjorl.2017.05.003

**Published:** 2017-06-01

**Authors:** Octavio Garaycochea, Raquel Manrique-Huarte, Manuel Manrique

**Affiliations:** Universidad de Navarra, Clínica Universidad de Navarra, Departamento de Otorrinolaringología, Pamplona, Spain

## Introduction

Presently cochlear implantation (CI) is a worldwide well-known procedure for the treatment of severe to profound hearing loss.^1^ New surgical techniques and technological upgrades in the past years have helped to decrease complications of this procedure, but they still exist, defying experienced surgeons and device manufacturers. Major complications are defined as events that need surgical intervention with reimplantation, such as wound infections, device extrusions, device failure and electrode misplacement.^2^ The incidence of electrode misplacement fluctuates between 0.2% and 5.8%, including both extracochlear and labyrinthine misplacements.^3^ Despite the many described ways to ensure proper electrode array positioning, there is no universally accepted protocol for intraoperative monitoring during cochlear implantation. We present a case of an intracochlear array misplacement (tip rollover) that was diagnosed intraoperatively with a fluoroscope after normal electrophysiological tests.

## Case report

A 9 year-old girl with good school performance came to the outpatient clinic due to hearing loss in the right ear of approximately two months with neither history of noise exposure or exposure to ototoxic substances. Physical examination was clinically normal and a pure tone audiometry revealed a profound sensorineural hearing loss of 100 dB of the right ear with normal function of the left ear. In the subsequent workup of the right ear a 0% speech discrimination was obtained in a speech audiometry (65 dB SPL in quiet); there was an absence of otoacustic emissions and stapedial reflex, and no response was obtained in auditory evoked potentials (click tones). Brain and temporal bone MRI was reported as normal.

**Figure 1:**
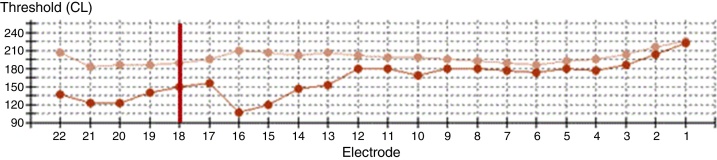
Intraoperative neural response telemetry: the first measure (tip rollover) is represented by the beige line and the second measure (reinsertion) by an orange line. The vertical red line marks the limit of the distal tip roll-over (e18–e22).

**Figure 2:**
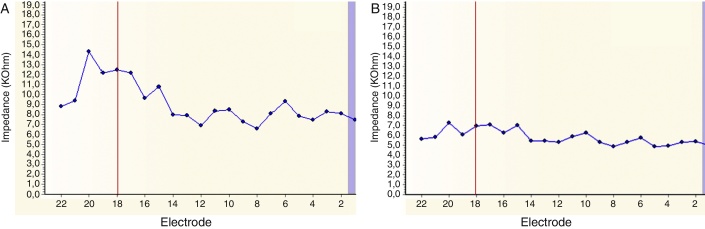
Intraoperative impedance. (A) The first measure (tip rollover); (B) second measure (reinsertion). The vertical red line marks the limit of the distal tip rollover (e18–e22).

**Figure 3:**
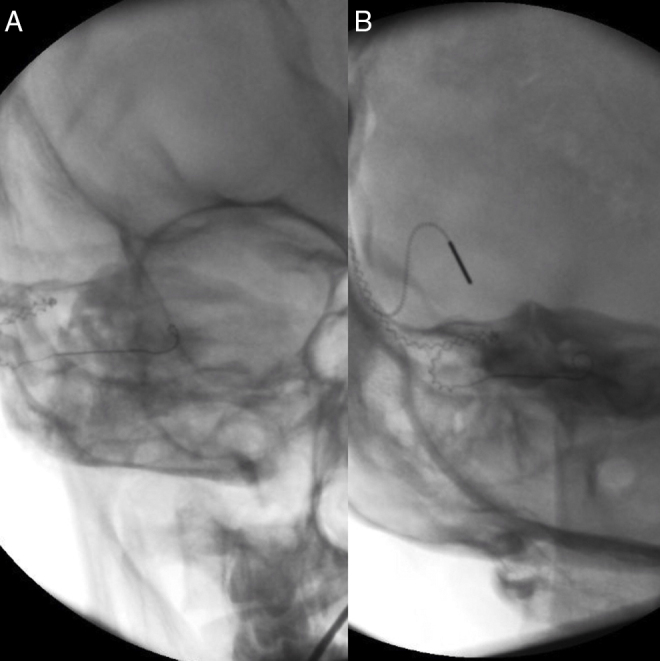
(A) Fluoroscopy after first insertion (tip rollover); (B) fluoroscopy after second insertion (correct placement).

The patient was diagnosed with single-side deafness and a cochlear implant was advised in the right ear. Surgery was performed via posterior tympanotomy, an extended round window cochleostomy performed and a CI532 perimodiolar array was inserted (Cochlear®). Intraoperative neural response telemetry (NRT) (Fig. 1) and impedance (Fig. 2) were first used to evaluate appropriate function using the CR220 Data viewerCochlear® and values in normal ranges were obtained. Then an intraoperative fluoroscopy was performed using an Arcadis® Orbic 3D C-arm, and even though the array was apparently well-inserted through the extended round window cochleostomy, the tip presented a rollover in its distal part, corresponding to the last five electrodes (e18–e22). The array was then removed and was repositioned in the insertor and reinserted for a second time. NRT and impedance were in normal ranges again, but when compared to the first measurement this time the impedance levels and the NRT thresholds had lower values. Correct insertion and shape of the cochlear implant array was visualized by intraoperative fluoroscopy (Fig. 3).

## Discussion

Intraoperative monitoring during CI to ensure proper electrode array position is still a subject of debate today. Electrophysiological testing based on neural response telemetry and impedance are still used for this purpose. NRT may suggest cochlear implant failure or malfunction,^3^ however the absence of NRT does not systematically indicate the lack of stimulation, because responses may be undetectable in some forms of auditory neuropathy. In the same way recording NRT does not necessarily imply correct intra-cochlear positioning of the implant because neural response telemetry can be obtained if the electrode is inserted in the vestibule or misplaced inside the cochlea.^4^ Normal impedance has been obtained as well in extracochlear and intracochlear arrays misplacements.^3^ Therefore some authors consider that there is no standard electrophysiological method of confirming CI placement.^3^ In our case, even though the impedance and the NRT of the first insertion were in the normal range of values, they had higher values compared with the measurement after the second and correct insertion. We presume that such findings suggest that the impedance of the last electrodes had higher values because the rollover of the array's tip generated more resistance to the electric signal, and that the NRT had higher values because the longer distance from the VIII nerve the more energy was needed to obtain a response.

Unlike electrophysiological testing which are normally used in all centres, an intraoperative radiologic control is not done routinely in all centres and its use is still controversial for some authors. Copeland et al.^5^ concluded that intraoperative plain radiographs had negligible value in assessing correct electrode array places, due to the low incidence of this complication and because the results would not significantly change the intraoperative management. Contrarily, Dirr,^6^ who recommends this technique, reported that although electrode misplacements were rare, nearly half of the cases of misplacement would have been diagnosed if an intraoperatively radiological study would have been done. In some centres, a plain X-ray of Stenver's trans-orbital view or a CT-scan is performed postoperatively and if a misplacement is diagnosed, a revision surgery is performed. In our centre we routinely make an intraoperative fluoroscopic study immediately after electrophysiological tests are made. We place the C-arm perpendicular to the modiolus, obtaining coronal images of the cochlea that help us diagnose possible misplacements, correcting them intraoperatively (like the case we present).

Extracochlear arrays misplacement is a rare complication (0.37%); the two most common anatomic sites reported are the superior semi-circular canal followed by the vestibule,^3^ but it has been described in other places like the middle-ear cavity, mastoid bowl, the petrous carotid canal or the Eustachian tube. In all cases misplacement requires revision surgery.^7^

Despite the fact that the incidence of intracochlear arrays misplacement is higher (scalar transition of the arrays, tip roll over, kinking or lopping),^8^ this type of misplacement is less known, probably because of the high rate of undiagnosed cases. There are several theories postulating what can cause this type misplacement such as increased force used by the surgeon or the possibility of obstruction to insertion. In our case we think that the cause was due an improper insertion of this new electrode. This particular electrode array is charged in a seal, following which it was manipulated by the “advance sheath”, and then the electrode was inserted in the cochlea bended since the beginning. Fortunately, the design of this device allowed us its reutilization and replacement with better impedance levels and the NRT thresholds. Nevertheless reinsertion is more traumatic to intracochlear structures.

Even though the impact of this type of misplacement is not as high as extra-cochlear type, it has been reported that minor arrays anomalies are related to poor results in CUNY speech scores, and in the particular case of array's tip rollover, it may lead to apical electrodes disability.^8^

## Conclusion

Electrophysiologic test alone is not enough to assure the correct insertion of the cochlear implant array in the intra-operative setting. Given the risk of a revision surgery due to an electrode array misplacement or when achieving a poor functional outcome of the cochlear implant during the follow-up, we consider that it is necessary to complement this study with a radiological one. Intraoperative radiological imaging gives real-time information about the electrode array location.

## Conflicts of interest

The authors declare no conflicts of interest.
